# Navigating Services: A Scoping Review of Mothers’ Experiences with Intimate Partner Violence and Social Systems in Canada and the United States

**DOI:** 10.1177/15248380251383935

**Published:** 2025-11-29

**Authors:** Eden Hoffer, Julia Yates, Tara Mantler

**Affiliations:** 1 Western University, London, ON, Canada

**Keywords:** intimate partner violence, mothers, social systems, child welfare, systemic harms, help-seeking, maternal responsibility

## Abstract

Intimate partner violence (IPV) has significant social, clinical, and public health consequences, particularly for mothers, who must navigate complex social service systems that may expose them to stigma, financial instability, and punitive system-based risks—including child apprehension. This scoping review explores how mothers with histories of IPV navigate social systems. Following the Joanna Briggs Institute framework and Mixed Methods Appraisal Tool quality assessment criteria, 5 databases were searched, and 13 studies met the inclusion criteria of peer-reviewed, scholarly journal articles, written in English, and conducted in Canada or the United States. Analysis revealed: (a) retributive consequences of help-seeking for mothers, (b) distrust of social systems by mothers who experienced IPV, and (c) disproportionate maternal responsibilities within IPV and child welfare involvement. Findings highlight a disconnect between social system responses and the support mothers require, punitive policies that discourage mothers’ help-seeking, and failure to address mothers’ caregiving burdens. Addressing these issues through survivor-centered, trauma- and violence-informed care-based reforms can foster healing environments rather than perpetuate harm.

Intimate partner violence (IPV) is a pervasive social, clinical, and public health concern affecting millions of women globally, with significant negative impacts on their physical and mental health and overall well-being (Sheridan & Nash, 2007; World Health Organization, 2012). According to the World Health Organization (2021), 30% of women aged 15 years and older have experienced IPV at some point in their lives, underscoring IPV’s widespread nature. IPV refers to actions within an intimate relationship that result in physical, psychological, or sexual harm, including physical aggression, sexual coercion, coercive control, emotional abuse, and controlling behaviors (World Health Organization, 2021). Although anyone can experience IPV regardless of their race, ethnicity, class, gender, or sexual orientation (Royal Canadian Mounted Police, 2019), the literature consistently demonstrates that IPV is gendered. Large-scale population studies show that men disproportionately perpetrate IPV, with women not only experiencing victimization at significantly higher rates but also facing greater psychological, physical, and economic consequences of this victimization (Cotter, 2021). This disparity is compounded by gendered power imbalances (McCarthy et al., 2018), societal norms (Shakya et al., 2022), and institutional responses to IPV (Osborn & Rajah, 2022), which influence how IPV is perpetrated, experienced, and addressed. These factors contribute to the systemic disadvantages women with IPV histories face when seeking support, such as housing insecurities, immigration and legal status challenges, stigmatization, and biases engrained in institutional responses, making it more difficult for them to escape cycles of violence and access the necessary care (Lieber & Escoda, 2016; McDonnell et al., 2011; Willie & Kershaw, 2019).

In this review, the term “social systems” refers to formal institutions and service sectors that interact with mothers experiencing IPV, including child welfare (CW), healthcare, and legal systems. These institutions/systems are often positioned as sources of support but can also contribute to systemic harm through practices such as surveillance, punitive interventions, and gendered and racialized biases. While some literature explores IPV-specific services such as shelters or school-based programs (see, e.g., De Koker et al., 2014; Graham et al., 2011; Yakubovich et al., 2022), this review focuses on broader systems that mothers more routinely engage with when experiencing IPV, namely CW, healthcare, and legal services.

Among women who experience violence, mothers are a particularly vulnerable population, as the violence they endure is intertwined with their caregiving responsibilities, shaping how they navigate both their own safety and their children’s safety and well-being. Mothers experiencing IPV carry a dual burden, managing the psychological and physical consequences of IPV while fulfilling caregiving duties. Specifically, mothers are confronted with providing emotional support, ensuring their children’s safety, and maintaining stability—often within social systems that fail to acknowledge or support these dual realities (McDonald-Harker, 2016). At times, this extends beyond a failure to support and shifts into blaming or penalizing them. One such example is when institutional responses do not recognize mothers’ resistance strategies, treating them as ‘failing’ in their child protection responsibilities instead of understanding how their efforts to safeguard their children also shape their survival strategies (Fogarty et al., 2022). The presence of children also complicates mothers’ help-seeking, as they face punitive interventions, heightened institutional surveillance, and misplaced accountability with respect to child protection—compounding systemic harms that disproportionately impact mothers with IPV histories compared to survivors who are not mothers.

Further, both Canada and the United States have active CW systems that intervene in cases involving children’s exposure to IPV. However, key jurisdictional differences shape how this exposure is treated. In several U.S. states, children’s exposure to IPV is explicitly defined as a form of child maltreatment, triggering mandatory reporting and often automatic involvement of child protective services (Child Welfare Information Gateway, 2019). While legal definitions vary somewhat by state, the overall approach is relatively consistent and prescriptive. In contrast, Canada’s CW responses are governed at the provincial and territorial levels, resulting in substantial variation. Although most provinces and territories now include IPV exposure within maltreatment statutes, the threshold for intervention differs. Some require evidence of emotional harm (e.g., Alberta), while others allow for intervention based on exposure or risk alone (e.g., Newfoundland and Labrador; MacMillan et al., 2013; Nixon et al., 2007). This variation can lead to inconsistent application in practice and discretionary decision-making among Canadian CW personnel (Veenstra & Keenan, 2017). These structural differences likely influence how mothers navigate CW systems, shaping their experiences and access to support.

Notably, then, the gap in the literature lies in understanding how social systems respond to these mothers and whether these responses help or hinder their efforts to navigate violence. This area of study is of particular importance because mothers who are experiencing IPV often interact with multiple social systems. Research shows that these interactions can further traumatize IPV survivors. This trauma can stem from previous IPV experiences (Gorde et al., 2004; Humphreys et al., 2001), past encounters with service providers like police (Dichter, 2013), early-life abuse or neglect (Widom et al., 2014), and intergenerational, systemic oppression (Kaye & Glecia, 2025). Survivors also frequently report feeling stigmatized, disbelieved, or blamed by service providers, particularly within legal and CW systems (Kaye & Glecia, 2025), which leads to further harm and serves as barrier to their obtaining support (Grace, 2019; Pollack et al., 2005). Since these interactions significantly shape survivors’ perceptions of safety, it is crucial to recognize the role that social systems play in their abilities to navigate IPV (Sullivan & Goodman, 2019). While existing research has examined service provider interactions with IPV survivors, little attention has been given to how mothers with IPV histories navigate these systems. The intersection of IPV and motherhood presents distinct and multi-faceted challenges, such as CW involvement, securing safety for both themselves and their children, and managing institutional barriers. Given the established negative consequences of these interactions—including the risk of child apprehension (Banda et al., 2024; DeVoe & Smith, 2003; Fleury-Steiner & Thompson Brady, 2011; Hughes et al., 2011, 2016; Johnson & Sullivan, 2008; K. Nixon, 2009; K. L. Nixon et al., 2013; Kaye & Glecia, 2025; Purvin, 2007; Zink et al., 2003), separation trauma following children’s apprehension (Broughton et al., 2024; DeVoe & Smith, 2003; Johnson & Sullivan, 2008; K. Nixon, 2009), a lack of trust in social institutions (Banda et al., 2024; DeVoe & Smith, 2003; Fleury-Steiner & Thompson Brady, 2011; Johnson & Sullivan, 2008; Kaye & Glecia, 2025), and legal and financial instability (Broughton et al., 2024; K. Nixon, 2009; Kaye & Glecia, 2025; Miller & Manzer, 2021; Purvin, 2007)—it is critical to map out the existing literature to clarify what is known and where gaps persist. This review aims to address this gap by (a) shedding light on the supports that are or are not available to assist mothers in navigating these challenges, and (b) exploring the experiences of mothers with IPV histories and their interactions with providers across various social systems, including police, healthcare providers, and CW personnel.

## Methodology

This scoping review followed the framework refined and standardized by the Joanna Briggs Institute (JBI; M. D. Peters et al., 2015; M. D. J. Peters et al., 2020), which builds upon the foundational methodology developed by Arksey and O’Malley (2005) and subsequently advanced by Levac et al. (2010). The JBI framework provides methodological guidance for conducting and reporting scoping reviews, including a clear process for developing research questions, identifying relevant studies, selecting studies based on predefined inclusion criteria, charting/extracting the data, and synthesizing and reporting results. This approach promotes/ensures transparency, methodological rigor, and alignment with best practices for evidence synthesis in health and social research (M. D. J. Peters et al., 2020). A scoping review is particularly effective for identifying the breadth, depth, and nature of research in complex fields (Mays, 2001 as cited in Gottlieb et al., 2021) such as IPV and exploring the unique challenges faced by mothers when engaging with both general and “parent-specific” social systems, allowing for the synthesis of diverse cross-discipline academic sources and highlighting key gaps in the literature in pursuit of informing future research priorities.

### Criteria for Considering Studies for Review

Studies were eligible for inclusion if they: (a) Reported on the direct experiences of mothers who had experienced IPV and interacted with social service systems/providers, (b) offered clear descriptions of the implications of these interactions for mothers (e.g., perceptions of services, systems, or personnel; consequences), (c) were published in peer-reviewed scholarly journals, (d) were written in English, and (e) were conducted in Canada or the United States.

### Search Strategy for Identification of Studies

The literature search was performed between September and December 2024 using the following electronic databases: the Cumulative Index to Nursing and Allied Health Literature (CINAHL), PsycINFO, PubMed, SCOPUS, and Web of Science. Citation chaining was also employed (see Appendix A for search strategy).

### Study Selection

One thousand nine hundred twelve studies were identified and after removing 461 duplicates, 20 articles met the criteria for full-text review. Following full-text screening, 13 studies were included in the final analysis. The seven articles excluded after full-text review did not meet the inclusion criteria due to insufficient focus on mothers or the absence of analysis on interactions with social service systems or those working in these systems. These reasons are specified further in Appendix B.

Although—consistent with the inclusion criteria—the majority of included studies only sampled mothers who were survivors of IPV, some studies included broader participant groups in tandem with mothers (e.g., Kaye and Glecia, 2025 included 30 women with IPV histories—24 who had children and 6 who did not). These studies were retained when their findings contributed to relevant insights into interactions between mothers and social service systems. In such cases, these findings were extracted separately for analysis where possible.

### Collating, Summarizing, and Reporting of Results

The following data were extracted from each included study: author(s), publication year, study location, population, method, purpose, study design, social service(s) explored, important results, and policy/practice implications (see Appendix C). Where available, data on IPV severity and related challenges such as housing instability, mental health, and substance use were noted during extraction to contextualize systemic responses. Three complementary analytic approaches were employed in this review to provide a comprehensive understanding of the data: (1) numerical analysis was used to summarize the characteristics of the included studies (e.g., publication year, location, sample size, IPV definitions) to describe the scope and distribution of the literature. (2) thematic analysis was conducted following Braun and Clarke’s (2006) six-phase approach: familiarization, initial coding, theme development, review, definition, and write-up, and (3) trustworthiness was established using criteria from Lincoln and Guba (1985), including credibility (e.g., using direct participant quotations to support theme development), dependability (e.g., using Excel to systematically track codes), and confirmability (e.g., ensuring that themes/conclusions are supported by excerpts from the data, avoiding over-interpretation).

Phase 1: Article review and familiarization consisted of the researchers reading each included article multiple times to deeply understand the data and identify significant patterns related to mothers’ experiences with social service systems and institutions. Phase 2: Data coding: Key concepts related to mothers’ experiences with IPV and social service systems were identified through inductive thematic analysis, allowing patterns and themes to emerge directly from the data without imposing a fixed coding framework. However, this process was informed by prior scholarship and existing literature, which provided knowledge that guided—but did not limit—coding. Codes were generated manually through close reading of the articles and organized using Excel to track and refine emerging categories across studies. Codes were iteratively reviewed and refined to support consistent theme development. Phase 3: Theme development: The codes were then organized into broader themes reflecting common barriers, supports, and challenges faced by mothers who have experienced IPV when engaging with social services. Phase 4: Theme review and refinement: Once these themes were reviewed for relevance to the research question, they were reassessed to ensure they accurately represented the data in the articles and were refined for clarity. Phase 5: Finalization of themes: After finalizing the themes, clear definitions and labels were assigned to capture their relevance to mothers’ IPV experiences and service interactions. Phase 6: Example selection and connection to research question: Where applicable, examples from the studies were selected to illustrate each theme, and the findings were then connected back to the research question (Braun & Clarke, 2006). Phase 7: Interrater reliability checks: To enhance the trustworthiness of the data, interrater reliability checks were conducted by both researchers, ensuring dependability and reducing potential bias (confirmability) in the coding and theme development process (Lincoln & Guba, 1985). Phase 8: Quality evaluation: A quality appraisal of the included studies was conducted using the Mixed Methods Appraisal Tool (MMAT; Hong et al., 2018), consistent with current best practices for scoping reviews. Use of the MMAT ensured transparent evaluation of the included studies.

## Results

### Numerical Analysis

#### Data Collection

All 13 studies employed qualitative methodologies. This may be due in part to our search strategy, which included terms such as “experience,” “perception,” and “voices”—keywords commonly associated with qualitative research (see Appendix A). As a result, the final sample was composed exclusively of qualitative studies. Individual interviews were employed in 11 studies (Broughton et al., 2024; Fleury-Steiner & Thompson Brady, 2011; Hughes et al., 2011, 2016; Johnson & Sullivan, 2008; K. Nixon, 2009; K. L. Nixon et al., 2013; Kaye & Glecia, 2025; Miller & Manzer, 2021; Purvin, 2007; Zink et al., 2003). Two studies employed focus groups (Banda et al., 2024; DeVoe & Smith, 2003).

#### Theoretical Positioning

The majority of studies did not identify their theoretical positioning. Of the four that did, one was feminist intersectionality (Broughton et al., 2024), one was narrative theory (Hughes et al., 2016), one was structured action theory/social entrapment theory (Johnson & Sullivan, 2008), and one was Indigenous feminist thought and decolonial theory (Kaye & Glecia, 2025).

#### Geographic Location and Participant Population

Seven studies were conducted in the United States (*n* = 7) and six in Canada (*n* = 6). Two studies (Banda et al., 2024; Purvin, 2007) specified that they included mothers, but did not specify how many. The remaining 11 studies represented 301 mothers (Broughton et al., 2024; DeVoe & Smith, 2003; Fleury-Steiner & Thompson Brady, 2011; Hughes et al., 2011, 2016; Johnson & Sullivan, 2008; K. Nixon, 2009; K. L. Nixon et al., 2013; Kaye & Glecia, 2025; Miller & Manzer, 2021; Zink et al., 2003).

##### Participants’ Ages

Of the 10 studies that reported age, the average age was 33 years, with a range from 15 to 59 years (Broughton et al., 2024; DeVoe & Smith, 2003; Fleury-Steiner & Thompson Brady, 2011; Hughes et al., 2011; Johnson & Sullivan, 2008; K. Nixon, 2009; K. L. Nixon et al., 2013; Kaye & Glecia, 2025; Purvin, 2007; Zink et al., 2003). One study reported that four participants were young mothers, but did not provide participants’ ages (Hughes et al., 2016).

##### Participants’ Races

Of the nine studies that provided data on race, the majority of participants were White, with 105 White women represented. Four studies had a higher share of White women (Fleury-Steiner & Thompson Brady, 2011; K. Nixon, 2009; K. L. Nixon et al., 2013; Miller & Manzer, 2021), while three studies had more diverse samples and White women were not the majority (DeVoe & Smith, 2003; Hughes et al., 2011, 2016). Two studies had an equal number of White (*n* = 10) and Black participants (*n* = 10; Johnson & Sullivan, 2008) or White (*n* = 16) and African American or mixed-race participants (*n* = 16; Zink et al., 2003). Broughton et al. (2024) reported on participants’ ethnicities rather than their races. Three studies did not report on race, or it was unclear (Banda et al., 2024; Kaye & Glecia, 2025; Purvin, 2007).

##### IPV Definitions and Related Challenges

All 13 studies discussed lived experiences of IPV, though definitions and descriptions of IPV varied across the literature. Five studies adopted broad definitions of IPV (or domestic violence)—self-reporting psychological, emotional, physical, sexual, and economic abuse (Broughton et al., 2024; K. Nixon, 2009; K. L. Nixon et al., 2013; Miller & Manzer, 2021; Purvin, 2007)—while two focused primarily on physical violence (Banda et al., 2024; Fleury-Steiner & Thompson Brady, 2011). Following participants’ experiences of IPV, authors in two studies (K. Nixon, 2009; K. L. Nixon et al., 2013) described many of these experiences as severe—including participants being choked, punched, kicked, or having a weapon used against them—although they were not formally categorized using a severity scale.

Co-occurring challenges were frequently reported. In at least eight studies, mental health concerns—including depression, anxiety, trauma, and post-traumatic stress disorder were discussed (Broughton et al., 2024; Fleury-Steiner & Thompson-Brady, 2011; Hughes et al., 2011, 2016; K. Nixon, 2009; K. L. Nixon et al., 2013; Miller & Manzer, 2021; Purvin, 2007). Substance use appeared in four studies—often as a coping mechanism for the violence (Hughes et al., 2011, 2016; Miller & Manzer, 2021)—though it was used as an exclusion criterion in one study (Johnson & Sullivan, 2008). Housing instability—including homelessness and shelter reliance—was noted in over half of the studies (Broughton et al., 2024; DeVoe & Smith, 2003; Hughes et al., 2016; K. Nixon, 2009; Purvin, 2007).

##### Histories of IPV

All 13 studies involved participants with IPV histories. Women in six studies continued to experience violence after separation (Banda et al., 2024), including death threats (Broughton et al., 2024; Fleury-Steiner & Thompson Brady, 2011), harassment (K. Nixon, 2009; K. L. Nixon et al., 2013), and coercive tactics including stalking behaviors (Miller & Manzer, 2021). Some participants detailed having ongoing contact with their abuser (Broughton et al., 2024; DeVoe & Smith, 2003), remaining in or returning to violent relationships (DeVoe & Smith, 2003; K. Nixon, 2009; K. L. Nixon et al., 2013), or continuing to cohabitate with an abusive partner (Broughton et al., 2024; K. Nixon, 2009; K. L. Nixon et al., 2013; Zink et al., 2003).

Eight studies highlighted the challenges of leaving an abusive relationship, with some women maintaining contact with the violent male partners due to factors such as limited social support or financial stability. Yet, inconsistencies in reporting left gaps in understanding the duration of abuse. Five studies detailed lengths of violent relationships, which ranged from 2 months to 28 years, with a mean of approximately 7.9 years (DeVoe & Smith, 2003; Johnson & Sullivan, 2008; Kaye & Glecia, 2025; Miller & Manzer, 2021; Zink et al., 2003).

##### Participants’ Financial Strain

Of the eight studies that provided data on financial difficulties, 160 participants reported incomes at or below the poverty line, receiving government assistance, or living in households below the federal poverty level (Broughton et al., 2024; DeVoe & Smith, 2003; Fleury-Steiner & Thompson-Brady, 2011; Hughes et al., 2011, 2016; K. Nixon, 2009; Purvin, 2007; Zink et al., 2003).

##### Children

While all studies included mothers, only two studies specified the exact number of children participants had, for a total of 63 children (K. Nixon, 2009; K. L. Nixon et al., 2013).

#### Social System(s) Explored

Six studies explored women’s interactions with the CW system (Fleury-Steiner & Thompson Brady, 2011; Hughes et al., 2011, 2016; Johnson & Sullivan, 2008; K. Nixon, 2009; K. L. Nixon et al., 2013). Nine studies mentioned interactions with other systems alone or in tandem with CW, eight studies highlighted interactions with police (Banda et al., 2024; Broughton et al., 2024; DeVoe & Smith, 2003; Hughes et al., 2016; Johnson & Sullivan, 2008; K. Nixon, 2009; Kaye & Glecia, 2025; Purvin, 2007), four mention the court system (Banda et al., 2024; DeVoe & Smith, 2003; Johnson & Sullivan, 2008) or “court actors” (Miller & Manzer, 2021, p. 4547), and four studies explored interactions with healthcare and mental health providers (Broughton et al., 2024; DeVoe & Smith, 2003; Purvin, 2007; Zink et al., 2003).

### Thematic Analysis

Thematic analysis revealed three key themes related to mothers’ experiences navigating social systems in the context of IPV: (a) Retributive consequences of help-seeking—seemingly unexpected ramifications of seeking help from social systems for IPV survivors, (b) distrust of social systems among mothers who have experienced IPV—the distrust that some IPV survivors have toward formal systems such as the criminal legal system, CW and other social services, and (c) maternal responsibility in the context of IPV and CW—the ways mothers often felt held accountable for their children’s safety in the context of the violence they are experiencing, as well as beliefs related to their violent former partner’s accountability.

#### Retributive Consequences of Help Seeking

Mothers with histories of IPV often described negative, retributive experiences when interacting with social systems and service providers in those systems. Ten studies detailed such encounters with child protective services and the CW system (Banda et al., 2024; Broughton et al., 2024; DeVoe & Smith, 2003; Fleury-Steiner & Thompson Brady, 2011; Hughes et al., 2011, 2016; K. Nixon, 2009; K. L. Nixon et al., 2013; Kaye & Glecia, 2025; Purvin, 2007). Some mothers detailed the seemingly lifelong nature of CW intervention, explaining that they experienced CW involvement in their childhood(s) and were unclear on why—now as mothers—they were still being subjected to CW surveillance (Hughes et al., 2016). Some participants shared the unjust nature of CW requirements and conditions—particularly those put on *them* as IPV survivors—to attend programming such as counseling (Hughes et al., 2011). In nine studies, participants shared how the nature of CW interventions not only made them feel misunderstood and unsupported, but that they felt these interventions directly caused harm to them and their children by treating them as “grossly neglectful mothers” (Johnson & Sullivan, 2008, p. 246). Some participants shared that they felt the involvement was less than beneficial, such one participant in K. Nixon’s (2009) study, who deemed CW involvement “unhelpful, intrusive, and punitive” (p. 1). Participants described feelings of revictimization as a consequence of CW involvement as well as increased levels of anxiety, frequently resulting in severe physical and mental health concerns (K. Nixon, 2009). In addition, mothers in multiple studies expressed concerns over the lack of knowledge about IPV among providers in the CW system (K. Nixon, 2009) and how this leads providers to not consider the impacts of removing children from the IPV survivor—their “protective parent” (Banda et al., 2024, p. 514). For example, one mother in K. Nixon’s (2009) study noted that with respect to the IPV/her situation involving violence, the CW worker “didn’t understand or she didn’t ask the right questions” (p. 8). This lack of knowledge among service providers underscores how the disconnect between women’s needs and their encounters with CW reflects systemic/institutional-level shortcomings in service provider training, rather than individual failings on the part of CW workers.

Despite seeking help for safety and support, their interactions frequently resulted in increased stress, further victimization, and, in some cases, even criminalization, illustrating a troubling contradiction where systems that are seemingly designed to assist IPV survivors instead perpetuated harm against them. For example, in some cases, law enforcement arrested *both* the IPV survivor and perpetrator, reinforcing feelings of survivor disempowerment and distrust (Broughton et al., 2024; Kaye & Glecia, 2025). Similarly, some CW interventions framed survivors as “negligent mothers” rather than as victims, leading to the removal of their children (DeVoe & Smith, 2003). As illustrated in Kaye and Glecia’s (2005) study, for Indigenous women, specific barriers including intergenerational trauma, systemic racism, and a lack of culturally competent care further compounded these challenges, making engagement with legal and social services particularly burdensome and leading to retributive consequences such as child apprehension. This highlights how systems designed to support IPV survivors often unintentionally create punitive outcomes, deepening distrust in institutions and reinforcing the fear that seeking help may lead to harm for them and/or their children instead of protection.

#### Social System Distrust

Another finding in this study was that participants’ distrust of social systems—understood as a deep lack of confidence in institutions such as CW, healthcare, law enforcement, and legal systems due to historical injustices—often shapes how IPV survivors interact with these systems. This distrust reinforces survivors’ reluctance or hesitance to engage with institutions, fearing that this may lead to harm rather than protection. Distrust toward social services among mothers with histories of IPV was often rooted in prior experiences. In seven studies, participants’ distrust was shaped by their personal histories, such as negative prior experiences with service providers (Banda et al, 2024; DeVoe & Smith, 2003; Fleury-Steiner & Thompson Brady, 2011; Hughes et al., 2011; Johnson & Sullivan, 2008; K. Nixon, 2009; Miller & Manzer, 2021) and/or from hearing about others’ negative experiences with services, particularly with CW (Fleury-Steiner & Thompson Brady, 2011) which created or reinforced perceptions of these services as intrusive, ineffective, or even violent (Kaye & Glecia, 2025). In eight studies, systemic distrust was created or reinforced when mothers sought help from social services, law enforcement, court actors, or healthcare professionals, only to be met with accusations of negligence or unfit parenting or to be deemed a “bad” mother (Kaye & Glecia, 2025, p. 6; Miller & Manzer, 2021, p. 4551) instead of being provided with the support they required (Banda et al., 2024; DeVoe & Smith, 2003; Hughes et al., 2016; Johnson & Sullivan, 2008; K. Nixon, 2009; K. L. Nixon et al., 2013; Kaye & Glecia, 2025; Miller & Manzer, 2021). These experiences not only solidified existing distrust of these systems but cemented women’s refusal to engage with them in the future (K. Nixon, 2009). This distrust was particularly evident in CW contexts, where fear of having children apprehended was a significant barrier to system engagement (DeVoe & Smith, 2003; Fleury-Steiner & Thompson Brady, 2011; Hughes et al., 2011; Kaye & Glecia, 2025). However, it is helpful to note that even for mothers who did not trust social services, when they received information from providers on what they could expect from the process/next steps, their distrust of the system was reduced (Johnson & Sullivan, 2008).

However, systemic distrust often fueled mothers’ hesitation to disclose the IPV to police, medical professionals, or CW due to the perceived consequences (DeVoe & Smith, 2003; K. Nixon, 2009; Zink et al., 2003). Some mothers had already had their children apprehended (Hughes et al., 2011, 2016; Johnson & Sullivan, 2008; K. L. Nixon et al., 2013; Kaye & Glecia, 2025) and vowed to never call the police again (DeVoe & Smith, 2003; K. Nixon, 2009), while others avoided disclosing the IPV—even to those they thought might be able to help them—such as their children’s physicians (Zink et al., 2003), staff at their children’s school (Hughes et al., 2016), or CW personnel (Hughes et al., 2016; Johnson & Sullivan, 2008) to prevent potential CW involvement/due to fear of their children being apprehended. The distrust was often exacerbated by systemic failures, such as law enforcement seemingly not “justifying” intervention to assist IPV survivors until the risk of femicide became imminent (Kaye & Glecia, 2025). This sense of institutional betrayal, compounded by systemic inaction, deepened women’s distrust of the very social service systems that claim to offer them support and protection.

#### Maternal Responsibility in the Context of IPV and CW

Of the eight studies that explored expectations of mothers specifically in the context of IPV and CW (*n* *=* 8), findings indicated that these expectations often carried an implicit or explicit assignment of maternal responsibility (DeVoe & Smith, 2003; Hughes et al., 2011, 2016; Johnson & Sullivan, 2008; K. Nixon, 2009; K. L. Nixon et al., 2013; Kaye & Glecia, 2025; Miller & Manzer, 2021). Mothers reported feeling blamed for their children’s exposure to IPV (K. Nixon, 2009) and held accountable by systems for the consequences, as one participant in DeVoe and Smith’s (2003) study noted, “Because we as mothers are responsible. Fathers are not responsible. We are held fully responsible for anything that happens to our children” (p. 289). In four studies, this maternal responsibility was (re)enforced through interactions with CW (Banda et al., 2024; Johnson & Sullivan, 2008; K. Nixon, 2009) or the court system (Miller & Manzer, 2021), where mothers felt that they were treated as negligent or as complicit in the violence rather than as survivors seeking protection for themselves and their children. For example, a participant involved with CW shared that *she* is “the one that’s being punished. Me and my kids are being punished for another person’s actions” (K. Nixon, 2009, p. 12). This was also exemplified by the Indigenous women in Kaye and Glecia’s (2025) study, as one participant stated, “they don’t recognize us as victims. . .they treat us like a perpetrator!” (p. 6), though this was in reference to the criminal legal system more broadly. Interestingly, K. Nixon’s (2009) study also highlights the disconnect between CW intervention and the reality of the IPV situation: although all four participants were the focus of CW interventions, none were charged with child abuse. This suggests a disconnect between the punitive treatment participant mothers received and the lack of evidence that they were responsible for the harm, pointing to systemic tendencies to blame mothers for violence perpetrated by others.

Indeed, some mothers felt there were multiple sets of rules in the context of IPV: one for male perpetrators involving seemingly diminished accountability, and one for mothers who have experienced victimization (DeVoe & Smith, 2003), with mothers receiving the “short end of the deal” (Johnson & Sullivan, 2008, p. 248). This was illustrated by participants when mothers who had experienced IPV detailed the extensive requirements placed on them to retain or regain custody of their children (Fleury-Steiner & Thompson-Brady, 2011), described by participants in Johnson and Sullivan’s (2008) study as things mothers had to do to “earn” their children back (p. 248)—none of which were required of the perpetrator (Hughes et al., 2011), even in cases where the perpetrator had joint custody of the children (Johnson & Sullivan, 2008). This relates to the theme of responsibility by highlighting a perceived disparity in how accountability is assigned in IPV cases, while also suggesting that male IPV perpetrators are subject to fewer responsibilities or less (if any) consequences for their actions, while mothers who experienced IPV may be held to stringent gendered maternal standards (Miller & Manzer, 2021).

#### Quality Assessment

The methodological quality of the included studies was assessed using both the JBI Critical Appraisal Checklists and the MMAT (Hong et al., 2018). The MMAT was selected because it provides a comprehensive framework for appraising qualitative and mixed methods research, while the JBI checklists offered study-design-specific criteria. Quality assessment was initially completed by a single reviewer, with a second reviewer independently assessing a subset of six studies (46%) to enhance reliability and transparency. Overall, the methodological quality of the included studies varied, ranging from 3/5 to 5/5. The majority demonstrated high methodological quality, with 9 of 13 studies scoring 4/5 or 5/5. Four studies showed some methodological weaknesses, such as limited transparency in how themes and categories were determined and a lack of information.

#### Trustworthiness

To ensure methodological rigor, Lincoln and Guba (1985) criteria for trustworthiness—credibility, transferability, dependability, and confirmability—were applied. Credibility was strengthened through a systematic screening process. The first reviewer screened all titles and abstracts, and once articles had been selected for full-text screening, a second reviewer reviewed them independently. Discrepancies were resolved through dialogue with a third reviewer. To ensure transferability, detailed descriptions of included studies are provided. Information on study settings, study designs, and participant demographics was documented, enabling readers to extrapolate the study’s findings to other populations or contexts. For dependability, structured data extraction was conducted and consistently followed across studies to minimize selective reporting and ensure consistency. Using a standardized approach, the extraction process was thoroughly documented and can be replicated in future reviews. To ensure confirmability, the analysis process was documented, including study selection and theme development. An inductive approach to thematic synthesis was applied, allowing themes to emerge from the data, which were substantiated by direct participant quotes from the studies. Applying these criteria strengthens the credibility, transferability, dependability, and confirmability of this review, reinforcing the rigor and reliability of its findings.

## Discussion

This scoping review explored how mothers with histories of IPV experience interactions with social systems in Canada and the United States, with critical findings summarized in Table 1. Findings revealed that these mothers often face punitive consequences when seeking help, experience widespread distrust of social systems, and endure disproportionate caregiving responsibilities—often without adequate support. This review highlights mother-specific challenges in the context of IPV, which are often overlooked in broader IPV scholarship, underscoring systemic failures and how social systems exhibit gaps in support or IPV survivors who are mothers or even perpetuate harms against them.

**Table 1.** Summary of Critical Findings.

Theme	Description	Evidence Across Studies
Retributive consequences of help-seeking	Seeking help often led to punitive outcomes including child apprehension, revictimization, or formal system entanglement, including criminalization.	Banda et al. (2024); Broughton et al. (2024); DeVoe and Smith (2003); Fleury-Steiner and Thompson Brady (2011); Hughes et al. (2011, 2016); K. Nixon (2009); K. L. Nixon et al. (2013); Kaye and Glecia (2025); Purvin (2007).
Distrust of social systems	Often rooted in past harms and systemic failures, distrust of systems frequently led mothers who were navigating violence to avoid disclosing it or seeking assistance, even in emergencies.	Banda et al. (2024); DeVoe and Smith (2003); Fleury-Steiner and Thompson Brady (2011); Hughes et al. (2011, 2016); Johnson and Sullivan (2008); K. Nixon (2009); Kaye and Glecia (2025); K. L. Nixon et al. (2013); Miller and Manzer (2021); Zink et al. (2003).
Gendered maternal responsibility	Mothers were held responsible for both protecting children and managing perpetrators’ violent behavior, while fathers were rarely held accountable for their actions.	Banda et al. (2024); DeVoe and Smith (2003); Fleury-Steiner and Thompson Brady (2011); Hughes et al. (2011, 2016); Johnson and Sullivan (2008); K. Nixon (2009); K. L. Nixon et al. (2013); Kaye and Glecia (2025); Miller and Manzer (2021).
Failure to recognize mothers’ protective strategies	Mothers’ efforts to protect children from the violence (e.g., defensive resistance, calling the police, “sexual bargaining” with their violent partner) were often disregarded or misinterpreted by social service systems and their representatives.	Broughton et al. (2024); DeVoe and Smith (2003); Hughes et al. (2016); Johnson and Sullivan (2008); K. Nixon (2009); Kaye and Glecia (2025); Miller and Manzer (2021); Zink et al. (2003).
Systemic ignorance of IPV dynamics	Lack of social service system provider understanding of IPV led to harmful interventions, mislabeling survivors as neglectful mothers, and overlooking coercive control dynamics.	Banda et al. (2024); Hughes et al. (2016); K. Nixon (2009).

*Note.* IPV = intimate partner violence.

### Gendered Maternal Expectations Within Systems

A central theme across the literature is the persistence of gendered maternal expectations within CW, healthcare, and legal systems. Despite evidence that men perpetrate the majority of IPV, institutional responses frequently focus on monitoring and disciplining mothers (Azzopardi, 2022; LaLiberte et al., 2010), rather than holding perpetrators accountable (Lapierre, 2010; Lapierre et al., 2026; Strega et al., 2008). Literature shows that CW providers not only hold mothers responsible for protecting their children in the context of IPV despite the violence they are experiencing, but also perceive them as responsible for perpetrators’ actions. This reinforces harmful gender norms and places mothers in impossible positions: expected to protect their children while navigating violence, often without institutional support or recognition of their efforts. Until systems meaningfully engage fathers and hold them accountable in the context of IPV, mothers will continue to bear the brunt of institutional scrutiny and punishment instead of being provided with support/assistance, undermining both their safety and that of their children.

### Distrust of Systems

This study’s finding that mothers with IPV histories often distrust social systems aligns with broader scholarship on institutional failures with respect to IPV, which fuels survivors’ lack of trust in these systems (Morse et al., 2012; Grace, 2019) and discourages future engagement with critical services (Fulford & Ford-Gilboe, 2004; Reisenhofer & Seibold, 2013). By specifically focusing on mothers, this study contributes to the existing literature by highlighting how gendered maternal expectations within systems uniquely exacerbate challenges for these mothers, further discouraging engagement with essential services. Various social systems such as emergency departments may treat symptoms/injuries exhibited by IPV survivors, but may not inquire about how these injuries occurred (Khatib & Sampsel, 2022).

Similarly, criminal legal system-based interventions for IPV often criminalize survivors through mandatory charging policies and dual arrests—as was the case in two of the included articles (Broughton et al., 2024; Kaye & Glecia, 2025)—punishing women who engage in defensive resistance rather than acknowledging their experiences as victims (Conners & Johnson, 2017; Dichter, 2013; Grace, 2019). Indeed, one of the participants in Broughton et al.’s (2024) study, who was arrested in tandem with her partner, shared, “. . .I fought back that night ‘cause I felt like I had to, so the police arrested us both. . .it was awful. . .” (p. 1526). The findings in this study align with system-based failures because it underscore the ways in which social systems—such as the healthcare system—fail to acknowledge or adequately address the root causes of trauma. This builds on previous scholarship, which has identified how healthcare providers often treat symptoms of IPV but may not acknowledge that they arose from IPV victimization, and thereby fail to address the underlying causes (Khatib & Sampsel, 2022), such as for women accessing perinatal care (Mirlashari et al., 2022). When healthcare providers misinterpret or fail to contextualize physical or mental health concerns as symptoms of previous or ongoing IPV, they miss a critical opportunity to intervene effectively—especially for women in the perinatal period (Mirlashari et al., 2022). This failure to contextualize IPV-related symptoms means that IPV survivors will likely not be provided with the necessary supports to help them escape the violence. Similarly, when the criminal legal system criminalizes women instead of supporting them (Brown, 2000; Dichter, 2013; Grace, 2019), survivors will likely be deterred from seeking assistance from social systems in the future, since they are cognizant that systems which are seemingly meant to assist them will, in practice, perpetuate further harm against them and their children (K. Nixon, 2009).

Mothers with histories of IPV often face blame, stigma, and systemic harms that erode their trust in social institutions—particularly CW and legal systems (Banda et al., 2024; DeVoe & Smith, 2003; Fleury-Steiner & Thompson Brady, 2011; Hughes et al., 2016; K. Nixon, 2009). This distrust may mirror the behaviors observed in Crager et al.’s (2003) study, where all IPV survivors who had negative police encounters said they would not seek police help again. For mothers, the stakes are particularly high. As K. Nixon (2009) documents, one mother explained: “I’m not calling you. . . ‘Cause you’ll just take my son!” (p. 11). This fear leaves mothers more vulnerable to ongoing violence, with fewer perceived options for support (K. Nixon, 2009).

### Failure to Protect

This study also identified a critical systemic failure: rather than holding male perpetrators of IPV accountable, systems often shift responsibility onto mothers, expecting them to protect their children while overlooking the protective strategies they employ (Broughton et al., 2004; DeVoe & Smith, 2003; Hughes et al., 2016; Johnson & Sullivan, 2008; K. Nixon, 2009; Kaye & Glecia, 2025; Miller & Manzer, 2021). These strategies—including contacting authorities (DeVoe & Smith, 2003; Zink et al., 2003), using defensive violence, and subtle acts of agency to regain or maintain limited control—are often unrecognized or misinterpreted by systems that fail to appreciate the complexities of mothering in violent contexts (Black et al., 2020; Haight et al., 2007; Hayes, 2013; Osborn & Rajah, 2022; Rajah & Osborn, 2022). Some mothers physically intervened to shield their children from the perpetrator or engaged in “sexual bargaining” with their violent partner, described by one participant as making “deals with the devil” (Miller & Manzer, 2021, p. 4551). Although mothers put their children at the forefront of every decision they make in the context of IPV (Broughton et al., 2024) and employ a wide range of strategies to protect their children from their partner’s violence (Miller & Manzer, 2021), these unrecognized efforts reveal how motherhood adds complexities to IPV survival, forcing mothers to navigate self-protection and the protection of their children, often without adequate support.

Importantly, national policy differences also may shape these dynamics. As outlined earlier, U.S. CW systems often operate under mandatory reporting laws that can trigger automatic involvement in cases where children are exposed to IPV—whereas Canadian systems apply more discretionary thresholds that vary by province. These structural differences likely shape how mothers perceive and engage with CW systems, influencing their levels of trust, fear of intervention, and willingness to seek assistance for themselves and their children.

### The Need for Trauma- and Violence-Informed Approaches in Systems

As summarized in Table 2, this study’s findings have several implications for policy, practice, and research. To address persistent systemic barriers, there is a pressing need for supportive, non-punitive responses across healthcare, legal, and CW systems. Specifically, trauma- and violence-informed care—which acknowledges systemic trauma and adopts a strengths-based approach—has shown promise in improving survivor engagement and reducing institutional harm (Scott & Jenney, 2023; Wathen & Mantler, 2022; Wathen & Varcoe, 2023). Additionally, perinatal and postpartum periods may offer early opportunities for meaningful intervention (Cizmeli et al., 2018; Jackson & Mantler, 2017; Mantler & Jackson, 2024; Mirlashari et al., 2022).

**Table 2.** Implications for Policy, Practice, and Research.

Domain	Implications
Policy	• Reform CW and legal systems to avoid punitive responses to intimate partner violence survivors. • Ensure trauma- and violence-informed policies across sectors. • Support non-disclosure-based access to services.
Practice	• Train providers in trauma- and violence-informed care. • Embed intimate partner violence advocates in CW and healthcare settings. • Improve cross-sector collaboration. • (e.g., between CW, housing, healthcare domains). • Recognize and support mothers’ protective and resistance strategies within the confines of violence. • Reduce gendered expectations of maternal responsibility.
Research	• Explore survivor experiences with CW and legal systems in more depth. • Develop and apply survivor-centered theoretical frameworks. • Examine how accountability is (mis)assigned in intimate partner violence-related interventions.

*Note.* CW = child welfare.

Cross-sector collaboration is also critical. Integrated service models—wherein CW, housing, healthcare, and IPV agencies coordinate care—can reduce service fragmentation and improve outcomes for mothers and their children. Embedding IPV advocates within child protective service teams or emergency departments is one promising strategy (see, e.g., Basheer et al., 2022).

Importantly, systems must ensure that supports are accessible without requiring women to formally disclose violence they have experienced or engage with the legal system—which many fear due to potential retaliation and the risk of child removal. These approaches could include flexible funding for emergency housing, confidential, secure mental health services, and/or parenting supports that do not pathologize mothers who are IPV victim-survivors.

## Limitations and Future Directions

This study is not without limitations. The exclusive focus in this review on Canada and the United States may limit the generalizability of findings to other contexts, and language restrictions to only include studies that were conducted or written in English may have resulted in the exclusion of relevant perspectives. Moreover, given the dominance of cross-sectional research in this domain, future studies should prioritize longitudinal study designs to assess the long-term impacts of social system encounters on IPV survivors. Further, there is a critical need for intersectional research that explicitly examines the experiences of racialized, 2SLGBTQ+, and IPV survivors residing in rural locations, as only two studies spoke how many women (*n* = 24) were living in rural areas (Broughton et al., 2024; Kaye & Glecia, 2025), despite other studies flagging it as a known service barrier in which residing in rural locations may preclude women who have experienced or are experiencing IPV from accessing shelters, legal advocacy, or support groups (Fleury-Steiner & Thompson Brady, 2011). Similarly, only one study discussed women’s sexualities, noting that at the time of the abusive relationship, all women identified as heterosexual (Miller & Manzer, 2021). One study notes that all participants (*n* = 20) reported that their abusive partner was a man, but does not specify if participants were heterosexual (Broughton et al., 2024). However, two studies highlighted the role of sexuality in the context of IPV, citing additional intersectional disadvantages (K. L. Nixon et al., 2013) and barriers to adequate service provision, wherein providers sometimes exhibited heterosexism or a lack of commitment to serving lesbian women, which can hinder access to necessary support for women in the context of IPV (Banda et al., 2024). This study’s findings underscore the need to further explore the experiences of IPV among specific populations of women, which is also explicitly raised in three studies (Banda et al., 2024; Fleury-Steiner & Thompson Brady, 2011; K. L. Nixon et al., 2013).

## Conclusion

Mothers with histories of IPV report retributive consequences of help-seeking, distrust in social systems, and encounter disproportionate expectations of maternal responsibility in the context of IPV, especially in contrast to the limited or nonexistent responsibilities that are placed on their violent male partners. To ensure meaningful change occurs, trauma- and violence-informed survivor-centered approaches must be implemented throughout healthcare, legal, and CW systems, since the fundamental findings of this study illustrate that current responses to IPV—especially for women who are mothers—are often disconnected from their needs and realities, thereby serving to compound the harm they have already experienced in the context of IPV instead of providing them with support. Prioritizing the safety, autonomy, and well-being of IPV survivors, fostering trust between survivors and service providers, and ensuring meaningful perpetrator engagement and accountability are all critical. Systemic and policy reforms that center survivors’ voices can help lead to more equitable, effective responses to IPV, assisting in mitigating the widespread negative impacts of IPV on mothers and their children.

## Figures and Tables

**Appendix B.**
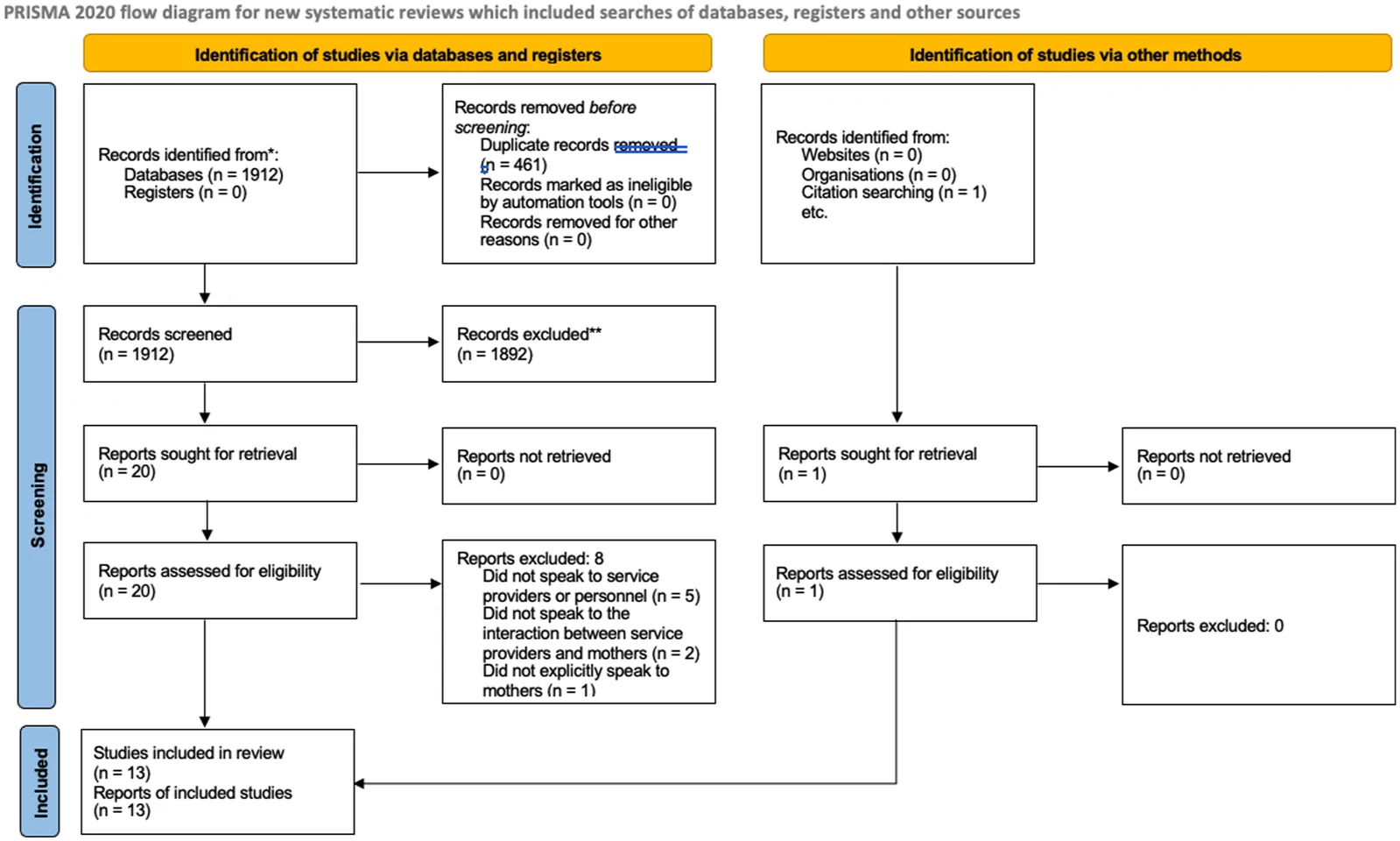
PRISMA-ScR. *Note.* PRISMA-ScR = Preferred Reporting Items for Systematic Reviews and Meta-Analyses extension for Scoping Reviews.

## Appendix A

### Search Strategy

The following four concepts were searched together using AND, with multiple terms for each concept grouped together using OR to ensure a comprehensive search.

1. “Intimate Partner Violence” OR “Domestic Violence” OR “Gender-Based Violence” OR “Wife Battering” OR “Spous* Abuse”
2. “Mother*” OR “Parent*” OR “Caregiver*”
3. “Child Protective Services” OR “Child Welfare” OR “Social Work*” OR “Social Welfare” OR “Police” OR “Law Enforcement” OR “Healthcare” OR “Health Personnel” OR “Emergency Medical Technicians” OR “Physician*” OR “Nurse*”
4. “Experience” OR “Perception” OR “Interaction” OR “Engagement” OR “Voices”

Cumulative Index to Nursing and Allied Health Literature (CINAHL) subject headings were utilized in CINAHL, PsycInfo OVID subject headings were used in PsycInfo OVID, and MeSH terms were used in PubMed.

**Appendix C.** Data Extraction Table—Navigating Services: A Scoping Review of Mothers’ Experiences with Intimate Partner Violence and Institutional Responses in Canada and the United States.

Citation	Study Location/Population	Purpose and Method	Demographics	Social System(s) Engaged	Important Results	Policy/Practice Implications	MMAT Score
Banda et al. (2024)	United States *N* = 140 families and professionals	To examine service delivery barriers for families experiencing DV and CW involvement via 16 in-person/remote listening sessions.	No demographic information provided.	CW, DV, and justice systems (police).	Four barriers identified: (a) communication issues, (b) inadequate services, (c) distrust in systems, and (d) poor service delivery.	Need for systemic, educational, and individual-level improvements—particularly within the CW system—to enhance collaboration and build trust with families. Emphasizes the need for fathers’ accountability where relevant.	3/5. This study is moderately methodologically strong, but it lacks some methodological transparency regarding coding verification and theme refinement—not enough detail is provided for these categories to be given a “yes” here.
Broughton et al. (2024)	Canada (Ontario, New Brunswick, and British Columbia) *N* = 20 mothers of children under 18 in IPV contexts	To explore mothers’ priorities in IPV, shaped by coercive control, inequities, and mothering assumptions, through semi-structured interviews	Age: Not specified Race: Predominantly 70% European descent Socioeconomic Status: 72% unemployed, 67% in financial difficulty, 20% not born in Canada (2 more recent immigrants, 10–11 years in Canada)	Police, healthcare, and DV agencies.	Mothers in IPV contexts prioritize children’s well-being, housing, and finances. However, structural barriers and system-based revictimization limit support access and increase stress, compounding victimization.	Calls for victim-centered services that address mothers’ competing priorities and systemic challenges in IPV contexts.	5/5. The study uses semi-structured interviews to explore women’s priorities in IPV contexts, analyzed using thematic analysis. The findings are well-supported by the data. This study is methodologically strong, making clear connections between data collection, analysis, and interpretation.
DeVoe and Smith (2003)	United States (New York) *N* = 43 mothers of children under 6 experiencing IPV	Explore help-seeking barriers among mothers experiencing IPV with children under 6 through focus groups.	Age: early 20s to mid-30s Race: 53% African American, 23% Latina, 12% multiracial, 5% Asian, 2% White. Socioeconomic status: avg. income $373/month, 50% no high school/GED.	Wide range of DV services—police, child protection, counselors, and shelters.	Mothers fear punitive consequences, like child removal, when reporting DV, leading to mistrust of social systems and underuse of services. They also faced a lack of appropriate services for young children exposed to DV.	Calls for better cross-system collaboration to support mothers and children in IPV contexts and reduce fears of child removal.	3/5. This study includes rich qualitative data, clear themes, and direct participant quotes in order to support claims. However, the coding process lacks transparency as it is not explained how codes and themes were refined.
Fleury-Steiner and Thompson (2011)	United States *N* = 19 mothers involved with child protective services due to IPV	Examine the role of resources and information in CPS investigations using in-person interviews.	Age: 21 to 45 (avg. 30.7), 37% Race: African American, 63% White Socioeconomic status: avg. income $956/month.	CPS, judicial system (i.e., police).	CPS workers often do not provide adequate information to mothers. While some received emotional and material support, gaps remain.	Recommends improved training for CPS workers to enhance communication and referrals, as well as better availability of community resources and coordinated responses.	4/5. This study is moderately methodologically strong and rigorous in some respects, involving rich qualitative data, clear themes, direct quotes/voices from participants. However, no explicit methodology is stated, making the methodological grounding somewhat unclear.
Hughes et al. (2016)	Canada (British Columbia and Manitoba) *N* = 64 mothers involved with CPS due to IPV	Explore the role of resources and information in CPS investigations using in-person interviews with narrative inquiry/analysis.	Race: 19 Aboriginal, 10 White women. Socioeconomic status: 24 women < $20,000/year.	CW system.	Women felt CPS ignored their circumstances (e.g., IPV, poverty, mental health) and often removed children without addressing root causes.	Calls for CPS to better understand mothers’ lived experiences, recognize their agency and strengths, and provide sufficient time and support for meaningful change.	3.5/5. This article effectively captured participants’ lived experiences and included compelling narratives and well-articulated themes but had methodological limitations including unclear coding processes.
Hughes et al. (2011)	Canada (British Columbia and Manitoba) *N* = 64 women involved with CPS due to IPV	Analyze the impact of CPS practices on women experiencing IPV, focusing on the support (or lack thereof) provided through interviews.	Age: 20 to 59 (avg. 36), 31 Race: Aboriginal, 6 Métis, 21 White, 4 Black. Socioeconomic status: most participants < $20,000/year.	Child protective services	CPS often placed responsibility on mothers—rather than addressing perpetrators’ behaviors. Services were often irrelevant or not helpful.	Emphasizes the need for CPS to provide concrete supports to IPV survivors, hold perpetrators accountable, and ensure ongoing support and communication for mothers.	4/5. This article involved a strong qualitative design with in-depth narratives, a well-documented recruitment strategy, and detailed use of NVivo. Potential weaknesses include limited coding transparency regarding how themes and categories were determined.
Johnson and Sullivan (2008)	United States *N* = 20 mothers with recent CPS interactions due to IPV	Examine perceptions of CPS interventions among mothers who are IPV survivors, focusing on the negative and positive impacts, through interviews.	Age: 21 to 52 (avg. 33). Race: 50% White, 50% Black. Socioeconomic status: income not specified.	Child protective services	Most participants felt blamed and misunderstood by CPS, with perpetrators avoiding accountability and mothers facing excessive, burdensome requirements.	Recommends empathetic, survivor-centered, trauma-informed CPS practices with a better understanding of IPV dynamics and equitable accountability for perpetrators.	5/5. This study demonstrates strong methodological rigor. It exhibits clear methodological choices, entails systematic data analysis, and well-substantiated interpretations of the data.
Kaye and Glecia (2025)	Canada *N* = 30 self-identified Indigenous women with prior experiences of IPV.	Examine the lived experiences of Indigenous women who sought systems meant to address intimate partner and gendered/sexualized violence through interviews.	Age: 22 to 57 (avg. 40.7) Location: 56.7% from Prairies, 24 mothers, 6 non-mothers.	CLS and CFS.	Indigenous women faced disbelief, victim-blaming, and criminalization within CLS and CFS, where perpetrators weaponized these systems, leading to child apprehension and further victimization, discouraging help-seeking.	Calls for decolonial, community-based, Indigenous-led approaches to IPV that center Indigenous women’s strength, agency, and sovereignty. This must be paired with system reforms in CFS and CLS to address victim-blaming, biases, and colonial violence, as current systems perpetuate settler-colonial harm and fail to support survivors effectively.	5/5. This study depicts strong methodological rigor, very clear theoretical grounding (Indigenous feminist and decolonial thought), and a transparent qualitative process. This is bolstered by trustworthiness measures to increase credibility (reflexivity, participant quotes, etc.). There is coherence between the research design, data collection, analysis, and ensuring validity.
Miller and Manzer (2021)	United States *N* = 25 mothers who have been out of abusive relationships for at least 1 year	Explore women’s efforts to protect children in IPV contexts and critique the civil court system’s failure in IPV cases through semi-structured interviews	Age: Mid-20s to early 70s. Race: 17 White, 6 Black, 1 Latina, 1 Asian participants, Education: from GED to doctoral degrees. Relationship length: avg. relationship length 9.25 years.	Court system (mediators, law enforcement, and judges).	Courts often overlooked IPV dynamics, favored perpetrators, and reinforced gender biases, resulting in poor outcomes for mothers and children.	Recommends IPV training for court personnel, trauma-informed approaches, and partnerships with domestic violence agencies.	5/5. This study meets all the MMAT qualitative criteria with a clear methodological approach, provides clear theoretical frameworks, and demonstrates strong alignment between data collection and data analysis, and well-supported findings.
K. Nixon (2009)	Canada (Alberta) *N* = 13 mothers involved with CPS due to IPV.	Analyze Alberta’s CPS policy and its impact on IPV-impacted mothers through qualitative interviews.	Age: 21 to 45 (avg. 34). Race: 11 Caucasian, 2 Aboriginal. Socioeconomic status: 5 earn < $20,000/year, 8 living in poverty.	Child protective services.	CPS intervention led to feelings of grief, identity loss, increased stress, and lacked focus on IPV dynamics. Children experienced detachment and poor school performance.	Advocates for tailored CPS services addressing IPV, including safety planning and a comprehensive assessment of individualized family needs.	5/5. This study demonstrates methodological rigor, well-structured data collection, and transparent data analysis.
K. L. Nixon et al. (2013)	Canada. *N* = 9 mothers with CPS involvement due to IPV.	Explore grief and loss among mothers with IPV experiences whose children were removed by CPS through interviews.	Age: 21 to 43 (avg. 32). Race: 7 Caucasian, 2 Aboriginal. Socioeconomic status: low income. Children: 28 children (ages 6 months–16 years).	Child protective services.	Child removal caused profound grief, loss of mothering identity, and significant health impacts. CPS often failed to provide concrete material or emotional support.	Advocates for CPS to prioritize mothers’ emotional and physical well-being, maintain communication about children’s welfare, and reduce practices that exacerbate grief and loss.	5/5. This study utilizes a clear methodology, grounded theory, engages in strong thematic analysis, and exhibits coherence between data collection and analysis/interpretation.
Purvin (2007)	United States (Boston) *N* = 54 low-income primary caregivers.	Examine how social welfare policies affect abused mothers’ ability to protect themselves and their children through ethnographic interviews and observations.	Age: 15 to 54 (mostly 20s–30s), Race: 1/3 African American, Latino, Euro-American, Education: varied, Socioeconomic status: half on welfare.	Examined a range of social services, including housing support, child services, frontline workers, and CPS.	Some policies mitigated IPV risks, while others increased participants’ vulnerability. The complex interplay between protective policies and those that unintentionally heightened risks highlighted the complexities of policy impacts.	Advocates for comprehensive policies that balance protection with mitigating unanticipated consequences for IPV survivors.	5/5. This study employs a rigorous ethnographic methodology, explicitly specifies multiple methods of data collection, and clarifies structured techniques for analysis.
Zink et al. (2003)	United States (Midwest) *N* = 32 mothers living in IPV shelters or participating in support groups.	Explore the role of children in mothers’ decision-making on IPV and their preferences for IPV screening and management in front of children through semi-structured interviews.	Age: 18 to 45 (avg. 32). Race: 50% White, 50% African American/mixed, Socioeconomic status: 75% below federal poverty level, Children: avg. 3 children.	CPS, children’s physicians.	Children often motivated mothers to seek help, either through their behavior or comments about the IPV. While some mothers wanted IPV screening and guidance from their children’s physicians, others feared revictimization and CPS involvement.	Calls for healthcare providers to adopt non-judgmental, trauma-informed approaches that balance IPV screening and education with sensitivity to mothers’ fears of judgment or punitive outcomes like CPS involvement.	3/5. This study does not explicitly state how themes were finalized beyond iterative coding, lacking transparency in the coding process and in theme validation.

*Note.* MMAT = Mixed Methods Appraisal Tool; CW = child welfare; DV = domestic violence; CLS = criminal legal system; CFS = child and family services; IPV = intimate partner violence.
